# The COVID‐19 pandemic and cause of death

**DOI:** 10.1111/1467-9566.13347

**Published:** 2021-07-13

**Authors:** David Armstrong

**Affiliations:** ^1^ School of Population Health and Environmental Sciences King's College London London UK

**Keywords:** cause of death, COVID‐19, death certificates, epidemics

## Abstract

This article explores the emergence and development of Death Certificates as a means of establishing the cause of death for individuals and populations. The difficulty in choosing which disease caused death when several are described on the Certificate explains why the number of COVID‐19‐related deaths has been difficult to determine. This problem also draws attention to the dominant biomedical explanation for the cause of death that both promote and circumscribe what can be recognised as a valid cause.

## INTRODUCTION

Studies of past cholera outbreaks (Hamlin, 2009; Rosenberg, 1962) have described the panic and flight associated with epidemics. Braudel, one of the foremost historians of the 20th century, claimed that epidemic disease in general induced profound disruptions of existing institutions such as markets, economies and socio‐political systems (Price‐Smith, 2009). Amongst sociologists, Strong has also argued that epidemics lead societies to get caught up in an ‘extraordinary emotional maelstrom which seems, at least for a time, to be beyond anyone's immediate control’ (Strong, 1990: 249). The COVID‐19 pandemic, however, seems different. The classical epidemics of the past such as bubonic plague, yellow fever or cholera had high mortality rates: plague in European cities, for example, often killed more than 20% of populations while cholera could kill up to 10% (Ranger & Slack, 1992). With this sort of impact, epidemics were very visible. Mortality from the COVID‐19 pandemic, however, is not high by the standards of past epidemics – though, as discussed below, the actual mortality rate remains a matter for debate. Further, social disruption has emerged not from the pandemic itself but from the response of central authorities in restricting social behaviour. And finally, the fear and panic that characterised the classical epidemic has not surged through the population as most have no direct experience of the epidemic's worst effects. In fact, there are many who challenge the supposed dangers of the virus and resist the social restrictions introduced to combat it (Romer & Jamieson, 2020).

The widespread resistance to the threat of the virus, whether through objections to lockdown or the flouting of public health measures or public protests against restrictions on individual behaviour, marks out COVID‐19 as an unusual epidemic. Indeed, COVID‐19 is an epidemic mainly known to the public through media reports that describe the numbers of cases, hospital and ICU admissions and the mortality rate. But while cases and hospitalisations might help map the extent of the pandemic without the spectre of death, these indicators would lose much of their significance.

The point of origin of mortality statistics for COVID‐19 is the Death Certificate that records what the patient died from. Ostensibly, the Death Certificate records the cause of death, yet its history, format and completion involve social judgements and processes. This article therefore examines the role of the Death Certificate in providing the lens through which the COVID‐19 pandemic came to be known and in doing so addresses the more general question of why people die. In part, the sociology of epidemics provides a context for this analysis, but it also occurs at the intersection of the sociology of official statistics and the sociology of (medical) record‐keeping.

Some of the earliest work on the social construction of official statistics (Kitsuse & Cicourel, 1963) recognised that reported rates of deviant behaviour were organisationally defined, classified and recorded. Since then, sociologists have approached official statistics with some scepticism, concerned to show how different rates of phenomena are determined, at least in part, by organisational practices (Hindess, 1973; Saetnan et al., 2011). Douglas (1966, 1967), for example, re‐analysed Durkheim's Suicide and showed how reported numbers were influenced by a variety of social factors, and Bloor et al., (1991) revealed a different perspective on routes of HIV transmission simply by presenting the data in an alternative format.

In similar vein, the sociology of medical record‐keeping has shown that written documents, usually in the form of case notes, mediate between health professional and patient. For example, MacIntyre (1978) observed how note‐keeping in obstetric clinics served to construct patients' identities and studies of the role of medical record‐keeping (Berg, 1992, 1996; Berg & Bowker, 1997; Heath, 1982) have shown how the process both objectifies the patient and reproduces the organisation of medicine. Yet while the Death Certificate is an important part of the process of constructing the official statistics of mortality rates and is based on a form of medical record‐keeping, it is concerned less with objectification (the patient is, after all, dead) or maintaining everyday clinical reality so much as making sense of death itself. Moreover, this narrative is dominated by rules and cultural assumptions that bring echoes of the 19th century into the 21st. In other words, unlike the immediacy of the clinical record or the contemporary biases in most official statistics, the Death Certificate and the accompanying rules for its translation into mortality statistics are determined by coding principles laid down almost two centuries ago that, though since amended, continue to dominate the interpretation of death. This account of the role of the Death Certificate therefore starts by considering classification principles and procedures in their historical context.

## DEATH CERTIFICATION

The Death Certificate first appeared in the mid to late 19th century across Western countries. This form recorded the name of the deceased, their age, gender, address, occupation and cause of death. In the preceding century, and earlier, some of this information had been recorded locally, often in ecclesiastical records, but the advent of a standard Death Certificate allowed the physical transmission of the data surrounding death to a central national repository where all deaths could be collated and population mortality ‘statistics’ compiled.

In part, the Death Certificate was a legal document giving official recognition that a person was deceased and their estate could be divided amongst any beneficiaries. But it was also a medical document in that unlike earlier local registers, it recorded the cause of death and was, in the main, completed by a physician. Even had a Death Certificate existed prior to the 19th century, it would have been impossible to ascribe a cause of death as death was then construed as a natural event, the limit of all living things. Death was the result of being old or of a Visitation from God. This explanation changed in the early 19th century with the spread of a theory that disease was brought about by a pathological lesion inside the body (Foucault, 1973). With the growth of this ‘pathological medicine’ and concurrent dissection of the body after death in the autopsy or post‐mortem, it became possible to identify the lesion inside the body that had brought about death. The Death Certificate affirmed and documented this new way of explaining and understanding death.

For the collation of mortality statistics, there needed to be an agreed list of possible causes for clinicians to use when completing the Death Certificate. An initial list was drawn up in the mid‐19th century in England by William Farr. This list then evolved as medical science identified new diseases and new causes of death. International consensus on a common list was only achieved at the end of the 19th century when, following a series of congresses, the International List of Causes of Death was drawn up with agreement it should be reviewed every 10 years to reflect changes in the classification of disease, particularly as a result of better diagnostic tools.

Agreeing to a common format for a Death Certificate that might record the cause of death proved more challenging. A patient with longstanding phthisis (later diagnosed as tuberculosis) might develop pneumonia and die. What had caused the death? Was it the phthisis that had so weakened the lungs that they were susceptible to infection? Or was it the pneumonia, without which death would not have occurred? For the certifying clinician, the question could be answered by entering both diseases on the Death Certificate as cause of death

in as few words as possible. When three or four causes have concurred in producing death, it will generally be sufficient to write them under each other without connecting verbs or particles. (Farr, 1842: 146)

But for the compilers of national mortality statistics, every death had to have a single cause so that the relative contributions of different diseases to population mortality could be determined.

There were two broad solutions to resolving the increasingly common problem of multiple causes of death. The first, pioneered in England, was to reformat the Death Certificate, inviting the certifying doctor to separate ‘primary’ from ‘secondary’ causes. This guidance, however, proved ambiguous: did primary and secondary refer to importance or position in time? In 1926, this was changed to ‘main’ and ‘contributory’ causes of death (Edge, 1928). In the United States, on the other hand, where Death Certificates varied in format (there were over 40 different ones in use in the early 20th century (Spain, 1923)), the task of choosing the cause of death was given to the coding clerks with the support of an Index of Joint Causes of Death that listed disease combinations together with instructions on which should be prioritised as ‘the’ cause of death.

**FIGURE 1:**
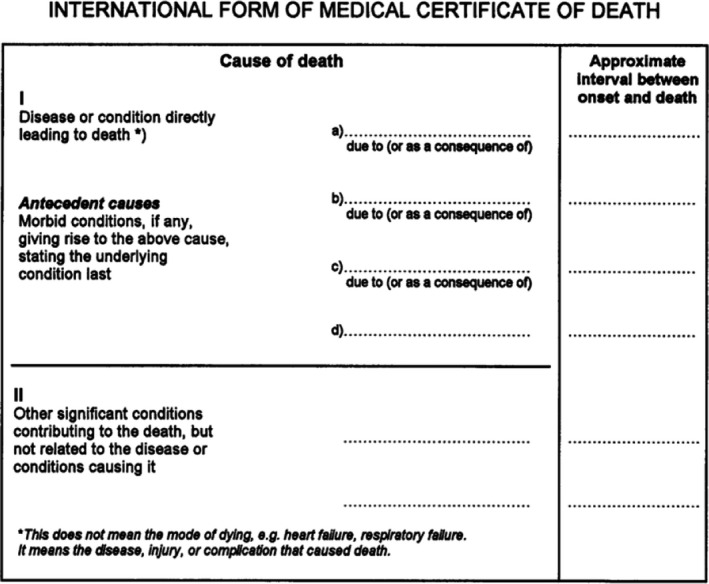
Death Certificate endorsed by WHO

Despite revisions during the early decades of the 20th century, the US manual of Joint Causes of Death proved increasingly unwieldy particularly as the potential number of causes of death in the International List increased in number (Dunn, 1936). By the middle of the 20th century, as the new World Health Organization took over responsibility for the International Lists of Diseases and Causes of Death, agreement was reached on a common Death Certificate to be completed by a medical practitioner that could be used by all countries. This Certificate had two sections (see Figure 1), one concerned with those medical conditions leading to death and the other to ‘Other significant conditions contributing to the death’. The first section described the disease or condition directly leading to death with three or four lines (at the discretion of different countries) underneath for preceding conditions so that the top line was always ‘due to’ or ‘as a consequence of’. The result was a cascade of causes as the clinical progression of disease was laid out in reverse order.

For coders, selecting the ‘cause of death’ then involved the application of various rules when more than one cause was described. The general principle was that the diagnosis on the lowest line was to be accepted if it could have given rise to all the conditions entered above it, that is the disease at the start of the train of events leading to death or the ‘underlying cause of death’. Three further selection rules gave the coding clerks discretion to over‐ride the clinician's report if there was a more credible sequence of events amongst the conditions listed on the Certificate. Coders, for example, could overrule the underlying cause on the Death Certificate if it was ‘highly improbable’ that the cause indicated by the physician was the starting point of the train of events leading directly to death’ (Faust & Dolman, 1965: 255).

There were then six further ‘modification rules’. These rules mainly addressed vague or imprecise reports of the underlying cause of death. Ill‐defined conditions, such as senility, frailty or ‘trivial conditions’ that might describe the overall context for the death, could be disregarded if a more specific cause of death was present on the certificate. This system allowed the clinician to exercise some judgement in completing the causal sequence on the Certificate, but the subsequent application of coding rules brought any idiosyncratic entries into line with agreed international custom.

## COVID‐19 DEATHS

Application of the standard death certification procedures – completion of the Certificate itself and identification of the ‘underlying cause’ as the cause of death – initially struggled to engage with the COVID‐19 pandemic of 2020. Not unexpectedly, in an era of multi‐morbidity most Death Certificates recorded more than one disease; in the United States, for example, 94% of COVID‐19 deaths had other conditions mentioned on the Death Certificate (Fineberg, 2020). Given that the ‘underlying cause of death’ was ‘the condition that began the chain of events that ultimately led to the person's death’ (Fineberg, 2020, citing CDC guidance), COVID‐19 at first seemed to have the same status as pneumonia, respiratory failure or cardiac arrest, the final endpoint in the train of events leading to death as a result of increased susceptibility to the virus of patients with other conditions. But from April 2020, the new rules (Kiang et al., 2020) required COVID‐19 to be the underlying cause, and therefore, the train of events was reorganised: any long‐term conditions, no matter how serious, were then relegated to Part II of the Death Certificate as ‘contributing’ causes.

Whatever the certifying physician's view of the train of events that led to death, the new coding rules ensured that COVID‐19 was the underlying cause of death if mentioned anywhere on the Death Certificate. With this procedure, the patient could be moved from the ‘diabetes’ or ‘heart disease’ column of causes of death (and thereby reducing the death rate for those diseases) to the COVID‐19 one. A patient dying from heart failure or cancer could become a victim of COVID‐19 even if just suspected:

COVID‐19 is an acceptable direct or underlying cause of death for the purposes of completing the Medical Certificate of Cause of Death … if before death the patient had symptoms typical of COVID‐19 infection, but the test result has not been received, it would be satisfactory to give ‘COVID‐19’ as the cause of death, and then share the test result when it becomes available. In the circumstances of there being no swab, it is satisfactory to apply clinical judgement. (ONS, 2020: 3)

Other approaches to identifying COVID‐19‐related deaths involved seeing whether there was any record of a positive COVID‐19 test in the patient's clinical notes or public health databases even though it was not described on the Death Certificate. Another common practice was to estimate the number of ‘excess’ deaths over the preceding five‐year period (Vandoros, 2020). This figure could only be an approximation as the death rate varies from year to year (not least because of influenza, another viral condition) and the fluctuations in death rates in 2020 without COVID‐19 could never be known. ‘Excess deaths’ was also just a summary statistic and did not enable the cause of death for the individual patient to be known.

The need to have a single underlying cause of death and application of the customary rules governing identification of the cascade of events that preceded death could have relegated COVID‐19 to a subsidiary position, if mentioned at all. For COVID‐19‐related deaths to be recognised, the usual rules for determining the underlying cause of death had to be ignored despite deaths occurring more commonly in those with other underlying conditions. Was the COVID‐19 virus the final blow or the underlying cause? Why did patients die during the COVID‐19 pandemic? There was no answer outside the trains of events on the death certificate as chosen by the clinician and the coding rules that sought to bring some order to the multiplicity of events preceding death.

## NOSOLOGY AND THE PANDEMIC

When Farr had assembled his original classification of causes of death in the mid‐19th century, he drew on disease classifications or nosologies from several earlier writers on the subject. From these, he identified three broad categories of causes of death. The first were epidemic diseases which had:

the peculiar character of suddenly attacking great numbers of people at intervals in unfavourable sanitary conditions … they have influenced not only the fate of cities, such as Athens and Florence, but of empires; they decimate armies, disable fleets; they take the lives of criminals that justice has not condemned; they redouble the dangers of crowded hospitals; they infest the habitations of the poor, and strike the artisan in his strength down from comfort into helpless poverty; they carry away the infant from the mother's breast, and the old man at the end of life. (Farr 1856: 76)

Farr called the second category ‘sporadic diseases’, those conditions that arose inside the body as pathological processes asserted themselves.

There is another vast, noiseless legion of diseases, marching at an even pace, neither exhibiting aggravation, nor creating sudden desolation, but never halting day nor night, and less under the control of external circumstances than epidemics. They are named sporadic diseases by medical writers, and are the ordinary maladies of every day occurrence. (Farr, 1839: 91)

In an earlier time, these might have been labelled as ‘natural deaths’ but under the new clinical medicine of the early 19th century they became pathological deaths.

The final category was those deaths due to external events such as ‘poisoning, asphyxia and injuries’. This classification captured those deaths judged as ‘unnatural’, that is before their natural time due to some, usually human, intervention. Violence headed the list, but it also included accidents and suicide. In such situations, the cause of death was clear. If a life had been terminated apparently prematurely, then the event that brought about that end was the obvious cause. This type of death had long been recognised as distinctive and had been dealt with through a medico‐legal system (such as the coroner's court in England and the medical examiner in the United States) to identify responsibility and any possible culpability in the death.

Deaths from violence and accidents superseded all other causes. Even if a patient had an underlying disease and perhaps complications of that condition meant death was imminent, this causal chain could be ignored if the patient died in, say, an accident. In the 20th century, it was recognised that this dominant position for Violent and Accidental Deaths ‘had never been quite satisfactory’ (Marshall, 1949: 293) and attempts were made to ensure that violence and accidents did not completely overrule pathological causes of death. Deaths could still be classified by the ‘underlying cause’, for example, if an accident had merely ‘accelerated’ the process, or the accident was a result of the disease (such as an epileptic fit) or if the injury was slight (perhaps an abrasion that later led to septicaemia) (Macphail, 1933). The Sixth Revision of the (ICD, 1948) tried to resolve the tension between accidents and pathology by introducing a dual classification, an ‘E‐Code’ for injuries from an external cause and an ‘N‐Code’ for the nature of the injury but the accident maintained its preeminent position when determining the single cause of death. Even so, the processing of these deaths within a medico‐legal system, as Timmermans (2006) has shown, marked a continuing tension between serving public health and criminal justice and between pathological explanations and moral agency.

In the same way, in principle, deaths from epidemics superseded deaths from pathological causes. A patient might have heart disease and be on a trajectory towards heart failure and death but if an epidemic disease suddenly intervened to bring that death forward then the epidemic disease was the cause of death. Deaths from both violence and epidemics therefore carried an implicit assumption about a ‘natural’ life span. Both epidemic diseases and violence ended life before its seeming proper time. It was the sudden and largely unpredictable act that gave both violence and epidemics priority in the rank orderings of causes of death. Of course, people eventually died; this understanding underpinned the large numbers of people who died from old age, senility or frailty, though many attempts were made to ‘pathologise’ these causes. In a way, any disease struck down life prematurely, and none more so than the result of violence and accidents or epidemics. The fear associated with epidemics seems rooted in both the violence to individuals and the violence to the social order (cf Strong, 1990).

The 19th century distinction between epidemic and sporadic (pathological) diseases implied different relationships between the inside and the outside of the body. Pestilence and other contagious diseases were clearly in the environment (and therefore of central concern for public health), invaded the body and increased the risk of death. Sporadic diseases, on the other hand, were essentially internal. They appeared within the body, grew, developed complications, led to other diseases and eventually brought about death. Epidemic diseases and those deaths caused by violence were both similar in as much as they involved external threats to the body that intervened in the natural order of internal corporal decay.

Determination of the cause of death in the influenza pandemic of 1918 presaged the problems with identifying COVID‐19‐related deaths in 2020. If a person with severe heart disease and influenza died, what should be their cause of death? The underlying problem was the need to identify a single cause of death: ‘The chaos caused by this arrangement during the 1918 influenza epidemic has probably not been quite realized’ (Roesle, 1926: 198). The difficulty was that the ‘Interpretations of such death rates necessarily should involve a consideration of the occurrence and influence of influenza as a contributory cause to other causes of death which were tabulated as primary’ (Dunn, 1936: 121). The result was that the influenza epidemic of 1918 had an effect on many other unrelated causes of death simply as a result of reclassification procedures (Dunn & Shackley, 1944). Later comparisons of influenza death rates across different countries (such as Spinney, 2017) had to assume certification processes were the same at a time when even the Death Certificate had not yet been standardised.

Despite the 1918 influenza epidemic carrying some priority in the contemporary ordering of causes of death, its subsequent less‐threatening annual occurrences were incorporated into a pathological framework. Should it be coded under ‘Infections’ or ‘Diseases of the Respiratory System’, and to what extent could it be construed as ‘underlying’? The Seventh Revision of the ICD, for example, gave priority to influenza when two or more conditions were entered on the Certificate without either being described as the underlying cause. The Eighth Revision then assigned those deaths to heart and circulatory disease and in effect removed influenza as the underlying cause (Klebba, 1975). One consequence was that without the priority accorded an epidemic disease, influenza deaths cannot simply be counted from Death Certificates and complex modelling is now used to estimate their ‘actual’ number (Reed et al., 2015).

The 19th century decision to separate epidemic, sporadic/pathological and violence as causes of death has continued to underpin the classification of death into the 21st century. These key elements can be identified in the chapters of ICD‐10: ‘It has stood the test of time and, though in some ways arbitrary, is still regarded as a more useful structure for general epidemiological purposes than any of the alternatives tested’ (ICD‐10, 2004: 10). Indeed, the response to the COVID‐19 pandemic relates to this old and fundamental distinction. On the one hand, the COVID‐19 virus produces an epidemic, a disease that comes from outside the body to attack it and sometimes kill it, an interruption to some ‘natural’ life span even in the very old or those with fatal diseases. On the other hand, the design of the Death Certificate was intended to eliminate the short‐term end‐of‐life processes, such as a rapid onset terminal pneumonia, in favour of more sustained long‐term ‘underlying’ conditions. Choosing COVID‐19 as a cause of death therefore reflects an old distinction that prioritises the unpredictable condition without a prior causal train of events; until, in time, a death within 30 days of a COVID‐19 diagnosis will no longer be registered as caused by the virus and the pathological domain will be restored.

## EXTENDING CAUSAL CHAINS

When constructing his classification of causes of death in the mid‐19th century, Farr drew attention to ‘external factors’ other than epidemics and violence: ‘Great numbers of the cases of disease in the previous classes are caused by external agents; by intemperance, cold, want, and effluvial poisons’ (Farr, 1842: 163). These external events were briefly filed under ‘Intemperance and starvation’ as a cause of death and included ‘Exposure to cold, and want of the necessaries of life’, ‘Starvation from cold’ and ‘Want of food and proper attention’. Many of these deaths were recorded as ‘natural death’ but, as Farr noted, ‘accelerated by’ want and privations (Farr, 1845).

With these comments, Farr recognised that the causal train of events leading to death extended beyond the pathological diagnoses that provided the centrepiece of clinical practice. Medicine, however, remained focussed on the body rather than the physical and social environment, and it was the body therefore that held the secret of death. Farr's observations on the effects of ‘want and privation’ simply pointed to the various pathologies that were their consequence. In the second half of the 20th century, however, these ‘external’ factors have assumed greater importance with the appreciation that health inequalities, for example, were rooted in factors outside the body and that health‐related behaviours lay on a causal pathway leading to ‘internal’ pathologies. Medicine, however, successfully accommodated these new explanatory variables in a discourse on risk factors, social determinants and multifactorial aetiology without disturbing the pathological core as displayed in the Death Certificate. The COVID‐19 pandemic brought these limits to what could be a legitimate cause of death into clearer focus. While it was quickly established that obesity, deprivation and ethnicity, for example, were important factors that increased susceptibility to the virus, these influences could not be designated as underlying causes.

Deaths from epidemic disease, as noted, had priority in the rank ordering of causes in that they unpredictably cut short the life span. At a population level, however, COVID‐19 was, in part, predictable. On the one hand, extensive ‘modelling’ sought to plot the pandemic's trajectory for weeks or months ahead as well as determine the risk profiles for many population subgroups. And whereas clean air, clean water and removal of effluent were the main goals of the 19th century public health movement, the key interventions for the new COVID‐19 pandemic were targeted at individual behaviour. Mixing with others, keeping social distance, wearing masks, etc., became important in keeping the epidemic under some sort of control. In effect, it was patterns of behaviour that allowed the pandemic to be predicted and individual behaviour that largely determined infection and death rates. When ascribing deaths to accidents, violence or suicide, medicine had recognised the role of human agency, but behaviour during the COVID‐19 pandemic could not be recorded on the Death Certificate as underlying or contributory causes.

While the content and format of the Death Certificate, especially what is a legitimate cause of death, continues to represent the core of the medical paradigm, the COVID‐19 pandemic has marked out the boundaries around that jurisdiction. It is what is excluded from the cause of death that says more about contemporary medicine than what can legitimately be included. The major risk factors for COVID‐19 are well‐established but most cannot be listed. Risk factors such as social determinants like deprivation and ethnicity are excluded as well as all those behaviours – assembling in crowded places, failing to self‐isolate, not maintaining social distances distancing, etc., – that enable the virus to spread. If one disease could delineate the boundaries of medical jurisdiction (as encoded in the Death Certificate), it is the COVID‐19 pandemic.

## WHY SOMEONE DIES

The COVID‐19 pandemic is caused by a coronavirus, SARS‐CoV‐2, but, as Arnold wrote, referring to earlier cholera epidemics:

Like any other disease, [it] has in itself no meaning: it is only a micro‐organism. It acquires meaning and significance from its human context, from the ways in which it infiltrates the lives of the people, from the reactions it provokes, and from the manner in which it gives expression to cultural and political values. (Arnold, 1986: 151)

The COVID‐19 pandemic is not different and understanding of it is refracted through a lens that takes account of its definitions and social consequences. It is the ‘man‐made images of pestilence’ that shape responses to the pandemic (Slack, 1992) which in their turn throw a light on the social order.

Emerging diseases are sources of instability, uncertainty and even crises that can make visible features of the social order ordinarily opaque to investigation. As societies respond to these challenges, features that we have taken for granted suddenly become transparent. (Dingwall et al., 2013: 167)

This new transparency has already appeared in areas such as health inequalities (Bambra et al., 2020; van Dorn et al., 2020; Herrick, 2020) and the ‘fractured society’ (Monaghan, 2020; Scambler, 2020). This article draws attention to another aspect of social life that has been thrown into sharp relief and that is the way death is classified and explained.

The ‘leading causes of death’ are routinely described in the medical literature (Woolf et al., 2021), and their mention has even become a standard feature of most medical sociology textbooks. There has, however, been some recognition that mortality statistics are ‘socially constructed’. Bloor (1991), for example, has shown the idiosyncratic ways in which clinicians complete Death Certificates while Prior (1989) has explored the social processes underlying the construction of mortality statistics in Belfast and foreshadowed several of the arguments of this paper. Further, elsewhere with Bloor he emphasised the key role of life‐tables.

The life table, then, is one of the most significant representations of life and death that our own culture has produced. It not only expresses a vision of life as a rationally calculable object, but also provides a set of background expectancies of normal, natural life spans. (Prior & Bloor, 1993: 356)

The struggle to identify the number of deaths from the COVID‐19 pandemic has further served to focus attention on why people die in the early 21st century.

Even at the best of times, identifying the underlying cause of death is an inexact science as evidenced by the many studies that show discrepancies between the Death Certificate and the post‐mortem/autopsy findings (Carter, 1985). But when it comes to COVID‐19 deaths, estimates vary, often considerably, as do estimates of the case‐fatality rate (Baud et al., 2020). Calculations of COVID‐19 mortality based on ‘excess deaths’ are constantly subject to further adjustment (Faust et al., 2021). Caduff (2020) has pointed out that it is impossible to calculate the case‐fatality rate accurately in the absence of systematic testing (that would provide a suitable denominator) but that calculation depends also on an accurate numerator, the number of deaths as a result of COVID‐19. This latter figure, however, depends on how the cause of death is determined. Mortality statistics, like the numbers of cases and hospitalisations, can allow the course of the epidemic to be plotted if the figures are collected using consistent methodology. Changes in methodology, or differences in rule application between different countries, however, mean that data become more difficult to interpret: ‘governments collect their statistics differently, making any cross‐border comparison, be it infections, recoveries, or deaths, imprecise’ (Moretti et al., 2021: 1–2).

The COVID‐19 mortality rate, just like other ‘official statistics’, shows how organisational classification and recording procedures underpin the reported numbers. At the same time, completion of the Death Certificate, however idiosyncratic, together with its format and accompanying interpretive rules constitutes a machinery with its own ‘social life’ (Savage, 2013) that is as much concerned with constructing a reality as measuring it. That reality is the explanatory role of pathology in explaining death over the last two centuries. Why did the heart stop, the brain wind down, the tissues start their slow decay? The older explanations of the appearance of the Grim Reaper or a Visitation from God were replaced with the certainty of pathological forces destroying life, a paradigm encoded in the Death Certificate. True, some older explanations survived, amongst them deaths from violence, accidents and suicide and the random ‘violence’ of an epidemic, but the causal train of pathological events as recorded on the Certificate told the essential story that underpins modern medicine. The process of death certification in the era of the COVID‐19 pandemic both exposes these core assumptions and circumscribes them with the limits of pathological explanation.

The pandemic has shown that determining why people die is not a simple task. The choice of an ‘underlying cause’ as specified on the Death Certificate fails to capture the myriad events and co‐morbidities that culminate in death. Everyone finally dies from cardiac arrest but attributing this cause to all deaths would be little better than recording every death as a Visitation from God. The certification question has thus become ‘why has the patient's heart stopped’? Gradually, medicine has opened for inspection of the chain of events that lead to that cardiac event. In so doing, medicine has affirmed its own explanatory framework around death. The contemporary answer to the question of why someone dies occupies its own paradigmatic space as much as did a ‘Visitation from God’.
